# An incidentally detected hepatic subcapsular hematoma in a very low birth weight newborn: a case report

**DOI:** 10.1186/1757-1626-3-32

**Published:** 2010-01-20

**Authors:** Hye Shin Ahn, Yun-Woo Chang, Dong Whan Lee, Kui Hyang Kwon, Seung Boo Yang

**Affiliations:** 1 Department of Radiology, Soonchunhyang University Hospital, 22 Daesakwan-gil, Yongsan-ku, Seoul 140-743, Korea; 2 Department of Pediatrics, Soonchunhyang University Hospital, 22 Daesakwan-gil, Yongsan-ku, Seoul 140-743, Korea; 3 Department of Radiology, Soonchunhyang Gumi Hospital, 250 Gongdan-dong, Gumi, Kyungbuk 730-030, Korea

## Abstract

**Introduction:**

A hepatic subcapsular hematoma in a neonate shows a non-specific presentation such as the presence of an abdominal mass without symptoms of hemorrhage and is clinically less distinguished as compared to cases detected during an autopsy.

**Case presentation:**

A neonate was delivered by vaginal delivery after 29 weeks and three days gestation with breech presentation. In a laboratory study, there were slightly increased levels of liver enzymes but the platelet count and hemoglobin level were normal. An abdomen ultrasonography and CT image demonstrated the cystic mass containing an internal thin septum with compression of the lateral margin of the right hepatic lobe and Morison's pouch. A CT image showed an irregular low-density lesion in the dome of liver that was suspected parenchymal laceration.

**Conclusion:**

We have described the sonographic and CT findings of an incidentally detected subcapsular hematoma of the liver in a neonate who showed a breech presentation, very low birth weight and was premature.

## Introduction

A subcapsular hematoma of the liver in a neonate may be uncommonly reported in clinical and imaging series as an asymptomatic or small size hematoma, although autopsy series have demonstrated an incidence rate of up to 15% [1-8]. This lesion should be suspected in infants with unexplained anemia or hypovolemia and in large infants and infants born by breech delivery [1-8]. We describe the sonographic and CT findings of a subcapsular hematoma of the liver in a neonate with nonspecific clinical features for a breech, very low birth and premature infant.

### Case presentation

A premature Korean female was delivered by vaginal delivery after 29 weeks and three days gestation to a mother with vaginal bleeding and premature labor with a breech presentation. The female patient had a difficult delivery and the patient developed a left clavicular fracture and cyanosis due to respiratory distress syndrome. The birth weight was 1420 g, and Apgar scores were 3 and 7 at one and five minutes, respectively. The infant was intubated shortly after birth with mechanical ventilation and respiratory distress syndrome had improved as seen on follow-up.

In a laboratory study performed at one day of age, there were increased levels of liver enzymes with an aspartate aminotransferase (AST) level of 164 U/L and alanine aminotransferase (ALT) level of 25 U/L and a total bilirubin level of 2.2 mg/dl, but the platelet count and hemoglobin level were normal. For the coagulation profile, the prothrombin time was prolonged at 15.6 seconds (normal range 9.9-13.1 seconds) and the active partial thromboplastin time was prolonged at 69.7 seconds (normal range, 23.5-37.5 seconds).

**Figure 1:**
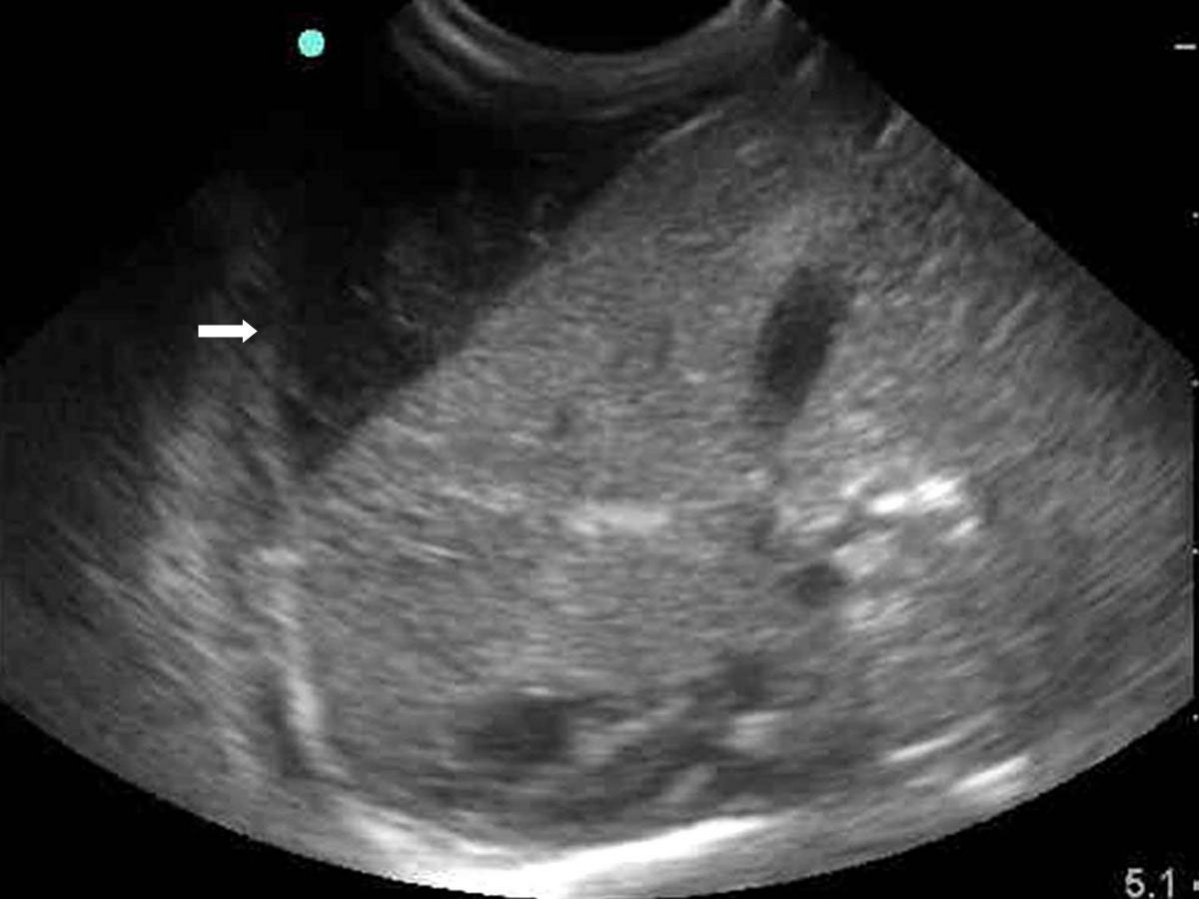
**Imaging findings are shown for a subcapsular hematoma in a one-day old infant**. An abdominal sonogram shows a cystic mass containing an internal thin septum with compression of the liver parenchyma in the right lobe of liver (arrow).

**Figure 2:**
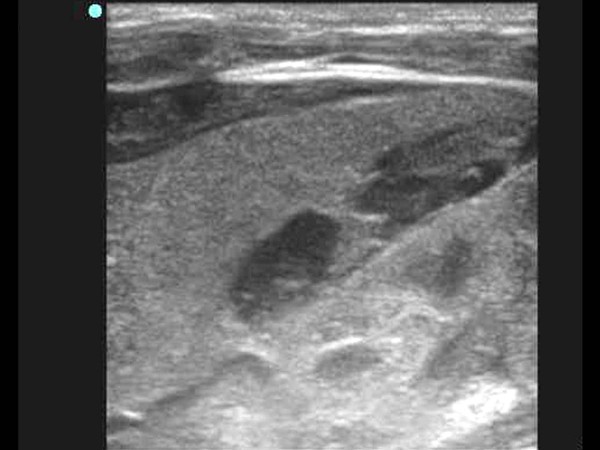
**A cystic mass with an internal septum is noted in Morrison's pouch**.

**Figure 3:**
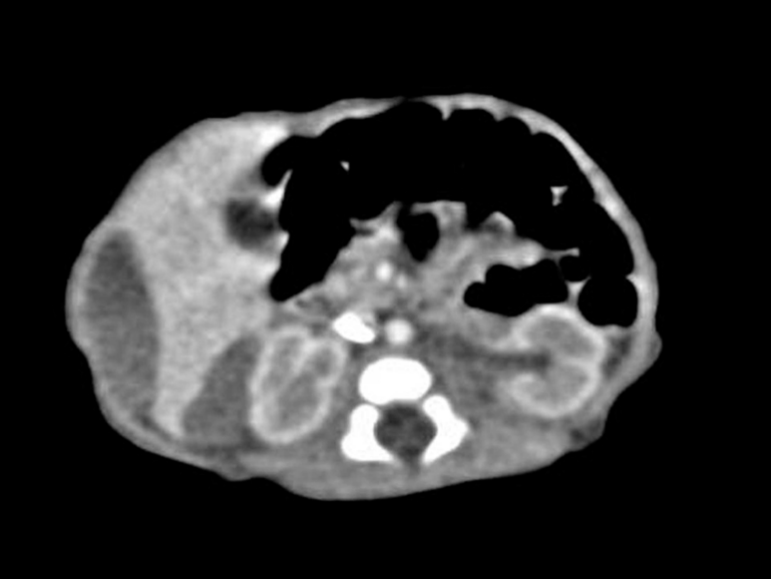
**A contrast-enhanced abdominal CT scan shows a well-defined, oval shape, non-enhancing, slightly low-density cystic mass in the subcapsular area of the lateral portion of the liver and hepatorenal space**.

**Figure 4:**
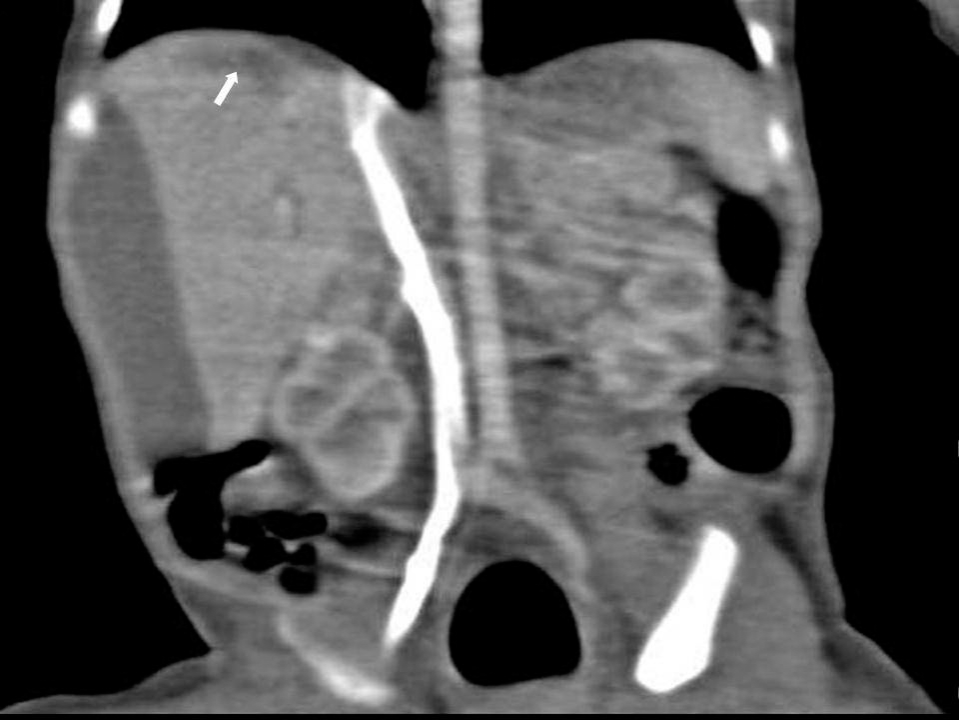
**A multi-planar reformatted coronal CT scan shows a partial linear low-density lesion in the dome of the liver presumed to be parenchymal laceration (arrow)**. An non-enhancing cystic mass is seen in the lateral margin of the liver with hepatic compression.

**Figure 5:**
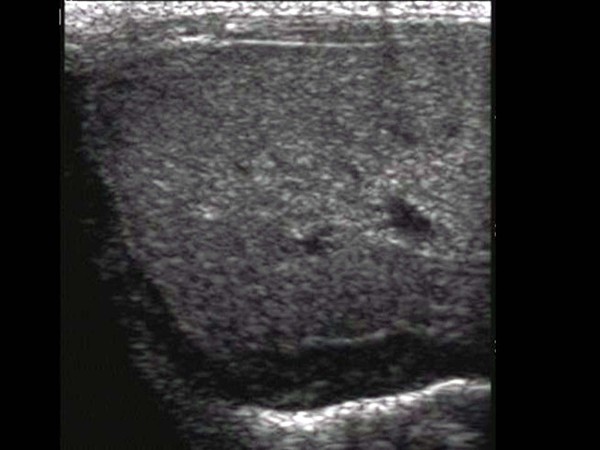
**A follow-up sonogram obtained after seven days reveals a markedly improved cystic lesion in the lateral portion of the liver**.

An abdomen ultrasonography examination was performed at two days of age and demonstrated the presence of an approximate 1.2 × 4.2 cm size cystic mass containing an internal thin septum with compression of the lateral margin of the right hepatic lobe. In addition, a cystic lesion in Morison's pouch was seen with the same features as that in the lateral area of the liver (Figure 1 and 2) Abdomen CT imaging was performed for the differential diagnosis including a subcapsular hematoma, liver abscess and panperitonitis. A CT image showed an approximate 3.1 × 1.0 cm size well defined, oval shape cystic mass in the lateral subcapsular area of the liver and the lesion compressed the adjacent liver parenchyma (Figure 3). An approximate 1.8 × 0.7 cm size cystic lesion was also noted in the area between the liver and the right kidney. An irregular low-density lesion in the dome of liver that was seen and parenchymal laceration was suspected. The CT finding was suspicious for a subcapsular hematoma because of the liver parenchymal laceration (Figure 4). A follow up ultrasonography examination was performed at seven days of age, and the previous noted cystic lesions adjacent to the liver and Morison's pouch had improved (Figure 5).

In a follow-up laboratory study, levels of liver enzymes had decreased but the level of total bilirubin had gradually increased and was 13.0 mg/dl at 27 days of age. Total bilirubin had decreased since 27 days of age. The platelet count decreased and was 8.2 mg/dl at 21 days of age. After transfusion of approximately 28 cc of blood, the platelet count gradually increased as seen in a follow-up study. The patient was discharged without abnormality.

## Discussion

A hepatic subcapsular hematoma in a neonate usually presents as a gradually increasing abdominal mass without symptoms of hemorrhage. Although autopsy series have demonstrated an incidence rate of up to 15%, the lesion is clinically less distinguished [1-8]. There are three major causes of hepatic injury in neonates, including obstetrical causes such as difficult labor, early travail and a breech presentation, neonatal causes such as hepatomegaly, coagulopathy, resuscitation, prematurity or post-maturity and maternal causes such as eclampsia and an elderly mother [1-8]. As was similar for the present case, a hepatic hematoma is common for premature infants and low birth weight infants with a breech presentation or thrombocytopenia as demonstrated in several cases. Many patients require management such as umbilical venous catheter insertion or mechanical ventilation and resuscitation that can cause a hepatic hematoma [3]. In a preterm infant, the abdominal configuration exposes more hepatic surface below the rib cage due to extramedullary hematopoiesis and liver laceration is induced by chest compression during delivery, which is due to stretching of attached multiple hepatic ligaments [1,3].

An abdominal sonogram shows non-specific findings and an intrahepatic hematoma can suggest a different diagnosis such as a liver abscess or hepatic mass [7,8]. In our case, cystic masses that contained multiple thin septa of the perihepatic area and Morison's pouch were noted on a sonogram. The presence of the masses and panperitonitis is required for the differential diagnosis. The usefulness of CT or MRI is disputable, but these imaging modalities may assist in the differential diagnosis [4,6-8]. A follow-up sonogram is helpful to confirm the diagnosis of a subcapsular hematoma and to identify a mass that has decreased in size [4,6,8]. Early detection of a subcapsular hematoma is important in an infant delivered via normal vaginal delivery with no significant clinical symptoms due to the difficulty of recognition of liver laceration. If a large amount of peritoneal bleeding progresses the bleeding can be fatal [4,6]. Conservative treatment is initially performed because of a great extent of a subcapsular hematoma is naturally absorbed.

We have described the sonographic and CT findings of an incidentally detected subcapsular hematoma of the liver in a neonate who showed a breech presentation, very low birth weight and was premature.

## Consent

Written informed consent was obtained from the patient for publication of this case report and accompanying images. A copy of the written consent is available for review by the journal's Editor-in-Chief.

## Competing interests

The authors declare that they have no competing interests.

## Authors' contributions

HSA analyzed and interpreted the patient data and the transplant. YWC was major contributor in writing the manuscript. DWL performed the clinical examination of the patient. KHK approved the final manuscript. SBY read and approved the final manuscript.
